# A Vibrotactile and Plantar Force Measurement-Based Biofeedback System: Paving the Way towards Wearable Balance-Improving Devices

**DOI:** 10.3390/s151229883

**Published:** 2015-12-15

**Authors:** Christina Zong-Hao Ma, Anson Hong-Ping Wan, Duo Wai-Chi Wong, Yong-Ping Zheng, Winson Chiu-Chun Lee

**Affiliations:** 1 Interdisciplinary Division of Biomedical Engineering, The Hong Kong Polytechnic University, Hong Kong, China; christina.ma@connect.polyu.hk (C.Z.-H.M.); anson.hp.wan@polyu.edu.hk (A.H.-P.W.); duo.wong@polyu.edu.hk (D.W.-C.W.); yongping.zheng@polyu.edu.hk (Y.P.-Z.); 2 Rehabilitation Engineering Research Institute, China Rehabilitation Research Center, Beijing 100068, China; 3 Institute of Active Ageing, The Hong Kong Polytechnic University, Hong Kong, China

**Keywords:** falls, postural stability, balance, sensory augmentation, wearable device, plantar force measurement, biofeedback, elderly

## Abstract

Although biofeedback systems have been used to improve balance with success, they were confined to hospital training applications. Little attempt has been made to investigate the use of in-shoe plantar force measurement and wireless technology to turn hospital training biofeedback systems into wearable devices. This research developed a wearable biofeedback system which detects body sway by analyzing the plantar force and provides users with the corresponding haptic cues. The effects of this system were evaluated in thirty young and elderly subjects with simulated reduced foot sensation. Subjects performed a Romberg test under three conditions: (1) no socks, system turned-off; (2) wearing five layers of socks, system turned-off; (3) wearing five layers of socks, and system turned-on. Degree of body sway was investigated by computing the center of pressure (COP) movement measured by a floor-mounted force platform. Plantar tactile sensation was evaluated using a monofilament test. Wearing multiple socks significantly decreased the plantar tactile sensory input (*p* < 0.05), and increased the COP parameters (*p* < 0.017), indicating increased postural sway. After turning on the biofeedback system, the COP parameters decreased significantly (*p* < 0.017). The positive results of this study should inspire future development of wearable plantar force-based biofeedback systems for improving balance in people with sensory deficits.

## 1. Introduction

Falls and consequent injuries have been and are major public health problems all over the world [1]. About 50% of young adults with long-term neurological conditions have experienced falls [2]. Approximately 30%–50% of people aged 65 or older living in the community, residential care facilities and nursing homes experience falls every year [1,3]. The incidences of falls and consequent injuries have been increasing along with the aging population [4]. The burden of the consequence of falls is heavy. They are associated with significant mortality and morbidity, reduced life span, reduction of quality of life, and huge hospitalization costs [5]. Risk factors of falls have been studied extensively. Balance and gait disorders are the second leading cause of falls, just coming after accidents [6]. Multiple factors contributed to balance and gait disorders, including aging, sensory abnormalities, musculoskeletal disorders, neurologic disorders, cardiovascular diseases, infectious and metabolic diseases, and psychiatric conditions [7]. In clinical practice, static balance is usually trained before dynamic balance.

Various balance training devices have been developed to improve static balance. Some of them induce physical challenges to balance by providing unstable support surface. Examples of these devices included wobble boards, ankle discs, balance sandals, foam pads, balance trampolines and tilting platforms, as reviewed in [8]. The balance challenges offered by these devices might not be suitable to people with moderate or severe balance disorders [8]. 

Some biofeedback systems, as reviewed in [9], have been developed in an attempt to improve the balance of patients with various types of balance disorders. The underlying principle of these devices is to improve balance by supplementing and enhancing somatosensory input [9]. Some systems measure the changes of plantar forces using a floor-mounted force-plate [10,11,12,13,14]. Some other systems mount inertial motion sensors (accelerometers and gyroscopes) on the user’s lower trunk or head to capture torso or head tilt in mediolateral and anteroposterior directions [15,16,17,18,19,20,21,22,23]. The sensors are wired to computers, which interpret body postures by processing the plantar force and body motion signals and send the corresponding control signals to a display (visual feedback) [24,25,26,27], an audio device (audio feedback) [13,14,28], or some type of vibrator (vibrotactile feedback) [16,19,21,22,23]. The feedback devices provide users with additional augmented sensory information on their body sway. Many biofeedback systems described in the literature were however only designed for use in laboratories and clinics. Patients would normally need to go to the clinics/laboratories to perform the balance training, which usually lasts for at least 2 weeks, as reviewed in [9].

In-home balance training contributes to the continuity and adherence of training [29]. Good compliance rates were achieved in home-based balance training programs [30,31], however, whether the training device is convenient to use could affect the compliance in patients. Large sensing/feedback elements and the need of a wired connection to a computer would discourage people from using the systems at homes. Making the biofeedback systems portable and convenient to use is necessary to allow them to be used at home. While current advanced technology enables microprocessors to be small and lightweight, which allows them to be wearable, little attempt has been made to turn hospital training biofeedback systems into wearable devices. Thin-film, in-shoe force sensors are ideal for the wearable purpose. They are obviously smaller and lighter than any force plates. In addition, it is possible for the thin-film sensors together with the associated electronic components for power supply, force analysis, and data transmission to be attached to the shoes. The replacement of inertial motion sensors with in-shoe force sensors would reduce the total weight of electronic components to be worn on the upper body. While some mobile in-shoe force measurement systems have been used to measure plantar pressure distribution with success as shown in [11,12,32,33,34], those devices did not provide real-time feedback of the changes in plantar forces to the users. In addition to force sensors, some studies have attached vibrators to the insoles [11,12,33]. Those vibrating insoles delivered stochastic resonance sub-threshold vibrations to the plantar surface of heel and the metatarsal heads upon floor contact, which was found to improve plantar sensation and balance [11,12,33]. However, the vibrating insoles led to pain and discomfort since the vibrators had to be made of rigid steel which produced excessive pressure on the metatarsal heads and heels [32]. With current wireless data transmission technology, consideration can be given to positioning the vibrators on other areas of the body while wirelessly maintaining the sensor connections.

The parameters accessing center of pressure (COP) excursions, which include mean distance and range, have been commonly used to evaluate postural stability during standing [35]. The ability to maintain good stability while standing still is a key indicator of fall risks [36]. Generally, increased COP movements are interpreted as an overall deterioration of postural stability [37], although a previous study found that other factors like anxiety could also increase of the COP displacements [38]. There was also evidence suggesting that better static balance performance is associated with better dynamic balance [35,39].

The objectives of this paper are: (1) to present a wearable biofeedback system, which measures and analyzes the changes in plantar forces and wirelessly sends control signals to vibrators located on the trunk and (2) to report the findings of an experiment conducted to evaluate the effects of the use of this system on static balance, assessed by measuring the COP movements of young and elderly people whose plantar tactile sensory input was experimentally reduced. This paper will also discuss some possible approaches for making the wearable device able to provide real-time postural feedback in dynamic situations. The ultimate goal is to pave the way towards the development of a wearable device which could improve balance in daily living.

## 2. Experimental Section

### 2.1. Participants

A convenience sampling approach was adopted to recruit thirty healthy subjects (including fifteen elderly adults aged over 65 years and fifteen young adults aged between 18 and 35 years). The young subjects were university students, and the elderly subjects were attendees at a local senior-citizen college. The sample size (a total of thirty subjects) produced a statistical power of 0.8, assuming a medium effect size of 0.5 and two-sided significant level of 0.05 on a two-way mixed-design ANOVA. Table 1 summarizes the subject information, including age, gender, height and weight. The subjects were fully independent, living in a community-based setting, and were capable of ambulation without assistive devices. Subjects with any neurological or vestibular disorders, diabetes, severe cardiovascular or pulmonary diseases, previous history of foot injury, foot deformity, amputation of the lower limbs, inability to attend the necessary re-evaluations, or inability to follow the instructions and procedures of the research protocol were not included in the study. This study was registered on the Chinese Clinical Trial Registry (ChiCTR-IPB-15006530, http://www.chictr.org.cn/ showprojen.aspx?proj=11141) and the Hong Kong Clinical Trial Registry (HKCTR-1853, http://www.hkclinicaltrials.com/). Ethical approval was granted from the authority of local university (Application Number: HSEARS20140211002). All participants signed an informed consent form after receiving oral and written descriptions of the research and the experimental procedures prior to the experiments. The subjects were aware that they could stop their participation if they encountered any kind of discomfort during the experiments.

**Table 1:** Subject Information.

[Mean ± SD]	Older Subjects (n = 15)	Young Subjects (n = 15)
Age (years)	70.1 ± 3.7	26.7 ± 2.9
Gender	6 females and 9 males	7 females and 8 males
Height (cm)	160.6 ± 7.6	167.6 ± 5.8
Weight (kg)	61.7 ± 11.4	61.4 ± 11.2

### 2.2. The Vibrotactile Biofeedback System

**Figure 1:**
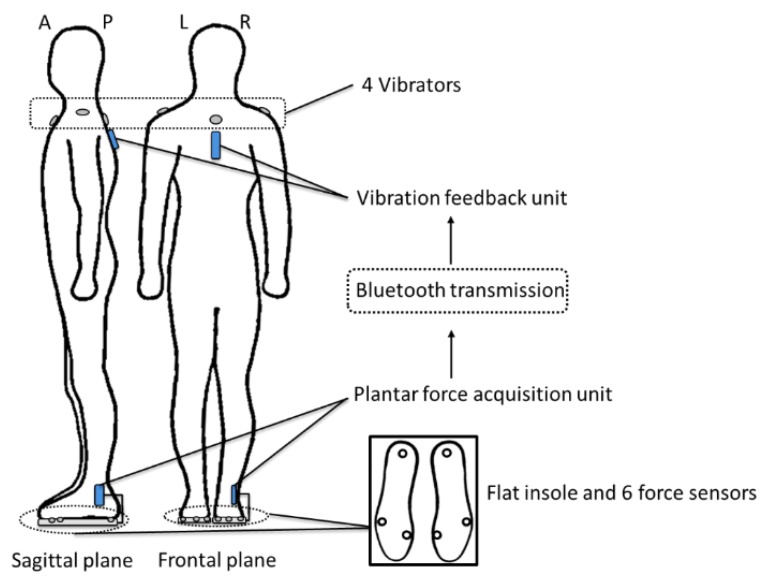
The vibrotactile system, consisted of a plantar force acquisition unit, a vibration feedback unit, four vibrators and six force sensors attached to a pair of flat insoles.

The vibrotactile biofeedback system consisted of two separate components of: (1) a plantar force acquisition unit and (2) a vibration feedback unit. The plantar force acquisition unit consisted of six thin-film force sensors (A301, Tekscan Co., Ltd, South Boston, MA, USA), a microprocessor unit (ATMEGA328P, Atmel Co., Ltd, San Jose, CA, USA), a wireless transmitter module (HC-05, HC Information Tech. Co., Ltd, Guangzhou, China), and a rechargeable lithium-ion battery (FLB-18650-3.0, UltraFire Co., Ltd, Shenzhen, China). The vibration feedback unit consisted of four vibrators (XY-B1027-DX, Xiongying Electronics Co., Ltd, Shanghai, China), a wireless receiver module (HC-05, HC information Tech. Co., Ltd.), and a rechargeable lithium-ion battery (FLB-18650-3.0, UltraFire Co., Ltd.). A total of six thin-film force sensors (25.4 mm × 14 mm × 0.203 mm, sensing area 9.53 mm diameter each) were attached to a pair of flat insoles with adhesive tapes. A series of insoles (2 mm thickness) with different sizes were manufactured by a certified orthotist using medium firm (30–35 Shore A Hardness) ethylene-vinyl acetate (EVA, Foot Specialist Footcare & Products Co. Ltd, Hong Kong, China). Four vibrators (10 mm diameter × 2.7 mm height each) were mounted at the anterior (the manubrium level), posterior (the first thoracic level), left (acromion) and right (acromion) side of subject’s upper trunk by adhesive tapes (Figure 1). Except for the force sensors, all electronic components in the plantar force acquisition unit were fastened to the lateral side of the lower leg by an elastic strap. The plantar force acquisition unit acquired the force data at the foot soles and delivered appropriate processed signals to the vibration feedback unit via the Bluetooth communication protocol. The vibration feedback unit activated the vibrators based on the processed vibrating signals. The vibration frequency and strength of the vibrator were 220 Hz and 1 G, respectively, which were found to be highly recognizable by humans [40]. Both sampling frequency and transmission rate were 10 Hz. The force sensors and vibrators were powered by the batteries in the plantar force acquisition unit and the vibration feedback unit. Rechargeable batteries with a capacity of 3000 mAh were used, which enabled the entire system to function continuously for 24 h.

Excluding the rechargeable batteries, the dimensions of the plantar force acquisition unit and vibration feedback unit were 4.0 cm × 1.5 cm × 1.7 cm, and 4.5 cm × 2.2 cm × 2.0 cm, respectively. The entire biofeedback system weighed less than 200 g, with the plantar force acquisition unit weighing 75 g and the vibration feedback unit weighing 78 g (including the high-capacity rechargeable batteries).

Force sensors were located at the heels, as well as the first and fifth metatarsal heads to monitor the subjects’ postural sway. The force sensors located at the first metatarsal head and the heel were used to detect the degree of anteroposterior body sway, and the force sensors located at the left and right fifth metatarsal heads were used to detect the degree of mediolateral body sway. The anatomical locations of heel, the first and fifth metatarsal heads were verified by a certified orthotist. Baseline readings were first collected while each of the subjects stood still with eyes-closed for 90 s in three repeated trials. The forces detected at each sensor across the 90 s in three test trials were averaged, respectively. The averaged values measured at each sensor were then multiplied by 110%, which were used to define the thresholds of allowable anteroposterior and mediolateral postural sway for each subject. A series of threshold-defining ratios (100%, 110%, and 120%) were tested in our pilot study, and the ratio of 110% produced the best outcomes in reducing postural sway.

Four vibrators providing vibrating stimulations were attached to the anatomical landmarks at the anterior (the manubrium level), posterior (the first thoracic level), left (acromion) and right (acromion) sides of upper trunk, which corresponded to anterior, posterior, left and right postural sway. Once the detected forces exceeded the preset thresholds, full magnitude of vibrations would be evoked at the corresponding vibrators. No vibrators were activated if the subjects’ measured forces were below the thresholds. 

### 2.3. Experimental Design and Procedures

All subjects were explained how the biofeedback system functioned prior to the experiments. They were informed that the vibration of each vibrator corresponded to one particular directional (forward, backward, left and right) body sway. They were instructed to move toward the opposite direction of the vibrator that had been activated. During the practice period, the subjects were instructed to incline forward, backward and laterally to experience the vibrations in four different directions to ensure that they understood the function of this system and were capable of using the fed back vibrations as a balance aid. The subjects were given 10 min to become familiar with the new biofeedback system. During the experimental period, a Romberg test was conducted to assess balance control [41]. When performing the Romberg test, the subjects were instructed to stand quietly on a force platform with feet together, with the arms crossed resting on opposite shoulders and eyes closed for 90 s for each trial. Such a balance test method and duration has high test-retest reliability [35]. Balance was evaluated under three experimental conditions: (1) without socks and the biofeedback system turned-off (condition 1); (2) wearing five layers of socks and the biofeedback system turned-off (condition 2); and (3) wearing five layers of socks and the biofeedback system turned-on (condition 3). 

Commercially available socks (Baleno Co. Ltd., Hong Kong, China) was used to simulate reduced plantar sensory input [42,43]. Mechanoreceptors at the plantar surface detect changes in plantar pressure distribution resulting from body motion, and initiate postural reflexes which help prompt the human body to more stable postures [44]. Previous studies have pointed that aging, diabetic malletis, and wearing socks reduced the cutaneous sensation of the plantar surface of the feet, and consequently led to balance disorders [42,43,45]. The socks were 1.6 mm thick, and made of 80.6% cotton, 16.7% polyamide and 2.7% Spandex. 

Each subject was randomly assigned to one of all possible sequences of three test conditions. Each test condition was repeated three times consecutively for each subject. Between two conditions, the subject was given a 10-min rest to eliminate any possible effect of fatigue. If the subject verbally reported any kind of discomfort during the experiment, the experiment would be stopped with the situation being recorded.

### 2.4. Outcome Measures

A force platform (OR6, Advanced Mechanical Technology, Inc., Watertown, MA, USA) was used to measure the relative location of COP signals to the coordinate origin of the force platform. The COP displacements were computed by the Nexus 1.8.1 software (Vicon Motion Systems Ltd., Oxford, UK), and then used to calculate the COP-based parameters using Microsoft Excel.

Based on the computed changes of locations of COP, the (1) mean distance (mm); (2) root mean square distance (mm); (3) the 95% confidence circle area (mm^2^); (4) the 95% confidence ellipse area (mm^2^); (5) planar diameters (mm); and (6) range in anteroposterior (AP) and mediolateral (ML) directions (mm) were calculated [37].

A 5.07/10 g Semmes-Weinstein monofilament (Connecticut Bio-instruments Inc., Bronx, NY, USA) was used to assess the plantar touch-pressure sensation of the feet with and without wearing the socks, which followed the standard testing procedures as specified in [46]. The dominant side was chosen for the testing, and determined by documenting the preferred leg for kicking a football [47]. The subjects were instructed to seat in a chair with their feet resting comfortably on a platform with their legs straight. Two applications and one sham application of monofilament pressing were performed at the plantar surface of the hallux, the first and fifth metatarsal heads. At each site, the monofilament was pressed vertically to the skin surface and bowed for at least 1 s. The testing consequences of three different sites and two different types of applications were randomized for each subject. The subjects were instructed to respond “yes/no” after each application. Scores were graded from 0 to 3 based on number of correct answers after three applications at each testing site [46]. The higher score of the monofilament testing represented the better sensation. The monofilament testing was performed on all subjects by the same examiner.

### 2.5. Statistical Analysis

Data analysis was performed using Statistical Package for Social Science (SPSS, version 21.0, IBM Corporation, Armonk, NY, USA). Descriptive analysis included mean and standard deviation values for demographic data. Wilcoxon Signed-Ranks Test was used to compare the monofilament score with and without wearing the socks. Two-way mixed-design ANOVA was used to assess if there were significant differences in all COP parameters (1) among the three different experimental conditions pooling all 30 subjects (effect of conditions, within-subjects effect); (2) between young and elderly subject groups pooling data of all three experimental conditions (effect of subject groups, between-subjects effect); and (3) among the three experimental conditions and the two subject groups (intervention effect). If the ANOVA indicated a significant difference, paired t-tests with Bonferroni corrections were used to perform multiple pair-wise comparisons among the three experimental conditions in elderly and young subject groups as well as combined, and independent-samples t-tests were performed to assess if there were significant differences between the young and elderly subject groups in each of the three experimental conditions. Further analysis regarding the degree of changes in postural sway upon using socks and biofeedback system was conducted by calculating the percentage differences in all recorded COP parameters between condition 2 and 1, as well as condition 3 and 2. Independent-samples t-tests were performed to assess if the percentage differences were significantly different between the young and the elderly subject groups. The level of significance was set at 0.05. Bonferroni corrections were performed to adjust the level of significance to 0.017 when performing multiple pair-wise comparisons. 

## 3. Results and Discussion

### 3.1. Results

As shown in Table 2, the average monofilament scores decreased significantly from 2.9 to 1.1 among elderly subjects (condition 2 *vs.* 1, *p* < 0.001), and decreased significantly from 3.0 to 1.0 among young subjects while wearing five layers of socks compared with the conditions of not wearing socks (condition 2 *vs.* 1, *p* < 0.001). 

**Table 2:** Comparison of monofilament scores with and without wearing socks.

Monofilament Scores (Mean ± SD)							
Elderly Subjects (n = 15)				Young Subjects (n = 15)			
Position	Without socks	With socks	*p*-value	Position	Without socks	With socks	*p*-value
Hallux	3.0 ± 0.0	1.1 ± 0.4	<0.001	Hallux	3.0 ± 0.0	1.0 ± 0.0	<0.001
1st metatarsal head	2.9 ± 0.3	1.0 ± 0.0	<0.001	1st metatarsal head	3.0 ± 0.0	1.0 ± 0.0	<0.001
5th metatarsal head	2.7 ± 0.8	1.0 ± 0.0	<0.001	5th metatarsal head	3.0 ± 0.0	1.0 ± 0.0	<0.001
Average	2.9 ± 0.4	1.0 ± 0.1	<0.001	Average	3.0 ± 0.0	1.0 ± 0.0	<0.001

NOTE: Higher monofilament score indicates better sensation.

**Figure 2:**
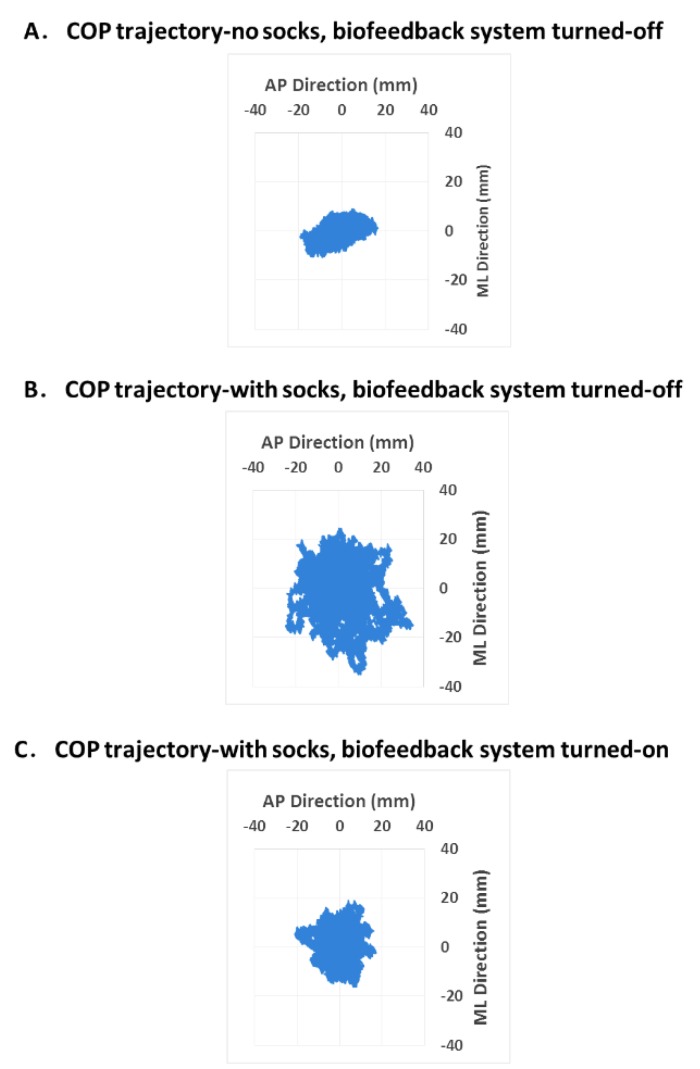
COP displacements in three different conditions in one elderly subject.

A typical example of the COP trajectory in all three experimental conditions of one elderly subject is shown in Figure 2. From the figure, it was clearly observed that the use of socks increased the excursion of center of pressure (condition 2 *vs*. 1). After the biofeedback system was turned on, with the socks continued to be worn, the excursion of center of pressure was reduced (condition 3 *vs*. 2). 

The results of two-way mixed-design ANOVA of the COP parameters revealed that there was a significant effect of experimental conditions (*p* < 0.05, within-subjects effect). No significant effect of subject groups (between-subjects), or the interaction among conditions and subject groups was found. The quantitative results of COP parameters pooling all 30 young and elderly subjects are displayed in Table 3. All COP parameters, including mean distance, root mean square distance, the 95% confidence circle area, the 95% confidence ellipse area, planar diameter, and ranges of COP in mediolateral and anteroposterior directions increased significantly while wearing socks (condition 2 *vs.* 1, *p* < 0.017). All COP parameters then decreased significantly after turning on the biofeedback system with the socks continued to be worn (condition 3 *vs*. 2, *p* < 0.017). No significant difference was found comparing condition 3 with condition 1.

Separate analyses of the young (n = 15) and elderly (n = 15) subject groups indicated that all COP parameters increased significantly after wearing socks in each of the two subject groups (condition 2 *vs*. 1, *p* < 0.017). Except for the anteroposterior range of COP in young and elderly subjects, all the other COP parameters decreased significantly in each of the two subject groups after turning on the biofeedback system with the socks continued to be worn (condition 3 *vs*. 2, *p* < 0.017). 

No significant difference was found comparing condition 3 with condition 1 in each of the two subject groups. Although no significant difference of COP parameters was found between young and elderly subject groups in each of the three test conditions, it was found that the percentage of increases from condition 1 to 2 in the elderly subject group were significantly higher than those of young subject group (elderly *vs.* young, *p* < 0.05) in each COP parameter. There was no significant difference between the young and elderly subject groups in percentage of decreases from condition 2 to 3. None of the subjects verbally reported any discomfort upon receiving the vibrating stimulations during the experiment.

**Table 3:** Comparison of COP parameters in three different conditions in all 30 subjects.

Young and Elderly Subjects (n = 30)				Condition 2 Minus Condition 1		Condition 3 Minus Condition 2	
COP parameters (mean ± SD)	No socks, biofeedback system turned-off (condition 1)	With socks, biofeedback system turned-off (condition 2)	With socks, biofeedback system turned-on (condition 3)	Difference	*p*-value	Difference	*p*-value
Mean Distance (mm)	6.68 ± 1.89	8.30 ± 1.94	7.24 ± 2.11	+24.2%	0.000	−12.7%	0.000
Root Mean Square Distance (mm)	7.67 ± 2.18	9.57 ± 2.29	8.26 ± 2.48	+24.8%	0.000	−13.7%	0.000
95% Confidence Circle Area (mm^2^)	568.12 ± 383.74	876.52 ± 516.33	660.86 ± 516.28	+54.3%	0.000	−24.6%	0.000
95% Confidence Ellipse Area (mm^2^)	550.47 ± 392.19	861.38 ± 490.55	668.10 ± 500.98	+56.5%	0.000	−22.4%	0.000
Planar Diameter- 95% Confidence Circle Area (mm)	25.69 ± 7.38	32.19 ± 7.93	27.50 ± 8.53	+25.3%	0.000	−14.6%	0.000
Planar Diameter- 95% Confidence Ellipse Area (mm)	25.20 ± 7.58	31.99 ± 7.85	27.73 ± 8.37	+26.9%	0.000	−13.3%	0.000
Mediolateral Range of COP (mm)	34.44 ± 11.60	46.91 ± 11.41	38.32 ± 9.38	+36.2%	0.000	−18.3%	0.000
Anteroposterior Range of COP (mm)	38.40 ± 10.00	45.84 ± 9.96	39.60 ± 12.09	+19.4%	0.000	−13.6%	0.001

NOTES: Mean Distance: represent the average distance from the mean COP; Root Mean Square Distance: Root mean square distance from the mean COP; Resultant Distance: the vector distance from the mean COP to each pair of points; 95% Confidence Circle Area: the area of a circle with a radius equal to the one-sided 95% confidence limit of the resultant distance time series; 95% Confidence Ellipse Area: the area of the 95% bivariate confidence ellipse, which is expected to enclose approximately 95% of the points on the COP path; Planar Diameter: the maximum distance between any two points of the area.

### 3.2. Discussion

This study developed a wearable biofeedback device providing vibrotactile feedback stimulations based on plantar force information. It was found that the device improved the static balance of subjects with experimentally simulated foot skin sensation deficits. With further optimizations of the device setting, this device might be used as a real-time mobile balance training device in daily life in the future.

The result of 5.07/10-g monofilament score is described as the best diagnostic criteria of loss of protective sensation, and a person who scored no more than 1 at any single site of the plantar surface of foot would be diagnosed with loss of protective sensation [46]. This study used multiple layers of socks to reduce the plantar tactile sensation of the subjects. The significant reduction in monofilament score indicated that the five layers of socks significantly compromised the plantar tactile sensory function of subjects, and potentially resembled the condition of loss of protective sensation over the foot which could happen in patients with diabetic neuropathy. Along with the significantly increased COP movements after using the socks (*p* < 0.017), the important contribution of plantar tactile sensation to postural stability is reinforced. This corroborates previous studies reporting increased postural sway after experimentally simulating impaired plantar cutaneous sensation through anesthesia [45], or reducing temperature of subjects’ feet [48]; as well as poorer dynamic balance performance in older adults while wearing socks [43,49]. When interpreting the findings of this study, attention should be paid to the fact that the plantar tactile sensitivity was experimentally reduced by the use of socks. Future studies should investigate if similar results occur in people with different physiological causes of balance problems, such as aging, loss of plantar foot tactile sensation, dysfunctions in vestibular, proprioceptive and visual systems, cardiovascular disease, metabolic disease, and muscle weakness [50,51,52].

Upon using the biofeedback system (with the socks still put on), the COP sway during quiet standing was significantly reduced. This suggested that subjects were able to take advantage of this vibrotactile biofeedback system to improve static postural stability. The finding of no significant difference between condition 1 and 3 implied that this system could help regain normal static postural stability in people who have compromised plantar sensory function. The underlying principle could be that the brain was able to integrate the biofeedback signals and natural senses to maintain static postural stability. Difficulty of keeping standing balance is associated with falls among older adults [53], and continued training is required to help them sustain good level of static balance [54]. This study determined the thresholds of allowable anteroposterior and mediolateral postural sway based on the averaged values of plantar forces measured during 3 trials of quiet standing of 90 seconds. Future studies may investigate the need of using different thresholds for people with various balance problems. In addition, people with balance problems may produce extreme force values, which may warrant the use of median force values throughout a period of standing rather than the averaged values when determining the threshold.

The significantly higher percentage changes in COP parameters after using five layers of socks in the elderly group, as compared to the young subject group, indicated that elderly subjects’ static balance performance was more sensitive to the reduction of plantar sensory input. The reason could be that older adults have degenerated muscle strength and other sensory functions due to aging, which are important to maintain balance control [5,55,56,57]. This may lead to increased postural instability during standing when one sensory input is experimentally reduced. Future studies are needed to verify the exact reasons.

Different methods have been used to improve sensation and hence improve balance. Examples are vibrating insoles, textured insoles, and biofeedback systems. The vibrating insoles and textured insoles provided vibrations and pressure stimulations to plantar surface of foot, respectively. As the vibrator was made of rigid steel, users complained of pain and discomfort when using the vibrating insoles [32]. It has been suggested that the economic and practicality problems associated with vibrating insoles may outweigh their beneficial effects on balance control [32]. At the same time, small semi-rigid plastic nubs or spikes on the surface of textured insoles tend to cause some pain and discomfort [58,59], and alter the normal gait patterns of the users [60]. Meanwhile, the device presented in this study is wearable and does not cause any discomfort. Future attempts can make good use of the wearable characteristic of this biofeedback device to improve dynamic balance during daily activities and allow balance training to be conducted anywhere. To this end, special attention should be paid to the use of sensors, feedback devices, and algorithm. 

The ability of attaching thin-film sensors to the shoes makes the devices wearable and reduces the total weight of electronic components worn on the upper body. The thin-film sensors could be inserted in the insoles, while the associated electronic components could be concealed either in the soles or vamps of shoes with proper design modifications. The sensor unit could be connected to the feedback module wirelessly. There are a couple of choices for feedback modalities, including vibrotactile [61,62], visual [24], and auditory [28]. However, visual and auditory feedbacks might not be appropriate if the device is used in daily life as they interfered with daily tasks of speaking, seeing and hearing [22]. Vibrotactile feedback eases the problem by providing tactile stimulations at users’ skin, as it does not hinder those daily tasks [61,62]. There may be concern if the feedback still works to improve balance if there are distractions from other activities. However, there was a previous study indicating that the positive effects of biofeedback on balance still persisted when the users were in high cognitive load (dual-task) situations [63]. This study attaches vibrators to the trunk. Although no discomfort was reported by the subjects regarding the skin-attached vibrators, future studies can identify the ideal locations of the vibrators for even better comfort and reception of feedback. Considering the cosmetic issue, the vibrators could be put at the trunk under the clothes and other body regions such as the waist and the wrist. Attempt could also be made to incorporate the vibrators into a smart-watch. 

It is feasible to make good use of the plantar force sensors to monitor the dynamic balance performance instantly. Walking with instability or on different terrains produces various force-time patterns [64,65]. In the future, the device can be configured by enhancing the algorithm to detect various physical and environment conditions and provide warning feedback in response to some conditions that may impose imbalance. Variable frequency and magnitude of vibration corresponding to the different input force signals could be adopted to distinguish the different physical and environment conditions. The device may not be able to initiate a response quick enough to prevent a fall that is about to happen. However, it can remind users of some conditions that would lead to imbalance. The plantar force data can be further used for real-time assessment of the gait and balance of the users. The computed real-time trajectory of COP could reflect the degree of lateral body sway during standing and walking, which was found to be a key indicator of fall [39]. In addition, it is also feasible to use plantar force sensors to obtain information about stance and swing time, double-limb support time, and weight distribution (or plantar force distribution) during walking, which are important parameters for detecting gait asymmetry and disorders [66]. All these inputs will provide innovative methods for real-time assessment of postural balance in activities of daily living, and inspires future possibility in the field of wearable devices for enhancing balance. 

## 4. Conclusions/Outlook

The immediate effect of a novel wearable vibrotactile biofeedback system on static postural stability improvement (estimated by measuring the COP displacements) was studied. The device provided corresponding directional information of body sway through vibrations delivered to the upper trunk, based on changes of plantar force detected by in-shoe force sensors at plantar surface of the feet. This study reveals that the device is effective in reducing the COP movements during standing in young and elderly subjects with experimentally induced reduction of plantar sensory input. The wearable characteristic and effectiveness of the device potentially allow static balance training to be conducted either indoors or outdoors in the future. With future enhancement of the algorithm in detecting dynamic instability during activities of daily living, it is possible for a wearable device to be able to work as a real-time balance aid in daily life. Further investigations and explorations are needed.
